# Supplementary figures and images for: Diversity of the Genomes and Neurotoxins of Strains of Clostridium botulinum Group I and Clostridium sporogenes Associated with Foodborne, Infant and Wound Botulism
Source: Toxins (Basel). 2020 Sep 11;12(9):586. doi: 10.3390/toxins12090586 (PMC7551954; doi:10.3390/toxins12090586)

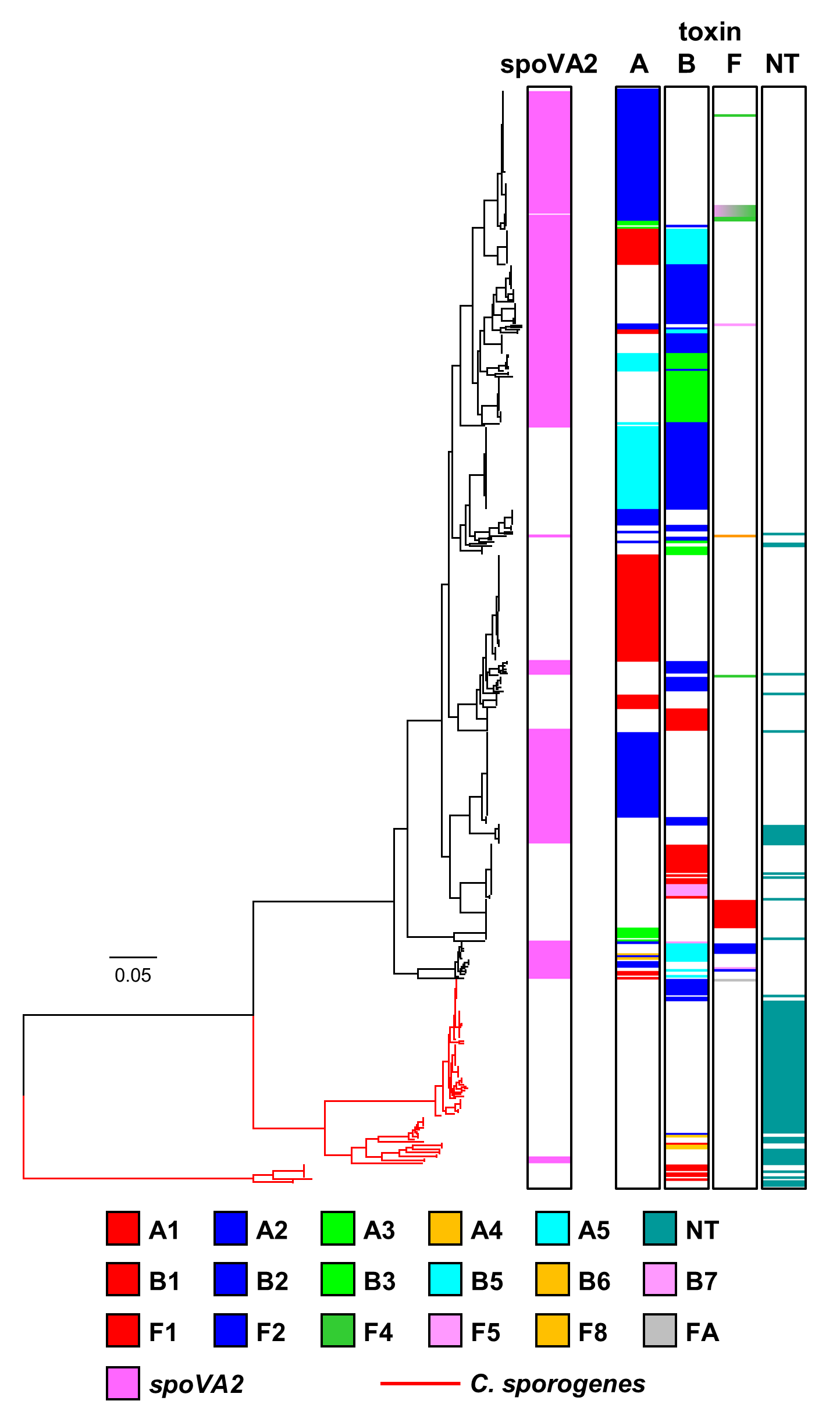

Supplement: Supplementary file 1 [file toxins-12-00586-s001.zip › toxins-904269-supplementary materials/Figure S1.tif]
